# EIF4EBP1 Overexpression Is Associated with Poor Survival and Disease Progression in Patients with Hepatocellular Carcinoma

**DOI:** 10.1371/journal.pone.0117493

**Published:** 2015-02-06

**Authors:** Yin-Lian Cha, Pin-Dong Li, Lin-Jing Yuan, Mei-Yin Zhang, Yao-Jun Zhang, Hui-Lan Rao, Hui-Zhong Zhang, X. F. Steven Zheng, Hui-Yun Wang

**Affiliations:** 1 State Key Laboratory of Oncology in Southern China, Sun Yat-Sen University Cancer Center, Guangzhou, Guangdong, China; 2 National Collaborative Innovation Center for Cancer Medicine, Sun Yat-Sen University Cancer Center, Guangzhou, Guangdong, China; 3 Department of Hepatobiliary Oncology, Sun Yat-Sen University Cancer Center, Guangzhou, Guangdong, China; 4 Department of Pathology, Sun Yat-Sen University Cancer Center, Guangzhou, Guangdong, China; 5 Rutgers Cancer Institute of New Jersey, Rutgers University, New Brunswick, New Jersey, United States of America; Medical University Graz, AUSTRIA

## Abstract

**Objective:**

EIF4EBP1 acts as a crucial effector in mTOR signaling pathway. Studies have suggested that EIF4EBP1 plays a critical role in carcinogenesis. However, the clinical significance and biological role of EIF4EBP1 in hepatocellular carcinoma (HCC) have not been elucidated. Therefore, we aimed to investigate the clinical significance of EIF4EBP1 in HCC.

**Methods:**

Total 128 cases of HCCs were included in this study. EIF4EBP1 expression in HCC tissues was detected by qRT-PCR, Western blot and immunohistochemistry, respectively. Then the relationships between EIF4EBP1 expression and clinical features as well as survival were analyzed.

**Results:**

The expression level of EIF4EBP1 mRNA is significantly higher in 60% (24/40) of fresh HCC tissues than that in the matched adjacent nontumor liver (NCL) tissues (P = 0.044). Similarly, EIF4EBP1 protein is notably upregulated in 8 HCC tissues (randomly selected from the 40 HCCs) measured by Western blot and is significantly increased in another 88 paraffin-embedded HCCs (53%, 47/88) by immunohistochemistry compared with the matched NCLs (P < 0.001). EIF4EBP1 protein expression in HCC tissues is significantly correlated with serum AFP (P = 0.003) and marginally significantly associated with pathological grade (P = 0.085), tumor number (P = 0.084), tumor embolus (P = 0.084) and capsulation (P = 0.073). Patients with higher EIF4EBP1 protein expression have a much worse 5-year overall survival (40.3% vs 73.6%) and 5-year disease-free survival (33.0% vs 49.0%) than those with low expression. Furthermore, Cox regression analysis shows that EIF4EBP1 protein is an independent prognostic factor for overall survival (HR, 2.285; 95% CI, 1.154–4.527; P = 0.018) and disease-free survival (HR, 1.901; 95% CI, 1.067–3.386; P = 0.029) in HCC patients.

**Conclusions:**

Our results demonstrate for the first time that EIF4EBP1 mRNA and protein are markedly up-regulated in HCC tissues, and the protein overexpression is significantly associated with poor survival and progression, which provide a potential new prognostic marker and therapeutic target for HCC patients.

## Introduction

Hepatocellular carcinoma (HCC) is one of the most prevalent malignant tumors, ranking as the third leading cause of cancer mortality worldwide [1]. An estimated 748,300 new liver cancer cases and 695,900 cancer deaths occurred worldwide in 2008 [2],and half of these cases and deaths took place in China. One of the reasons for the high mortality is that most patients are asymptomatic until the cancer is so advanced that the tumor cannot be treated with radical hepatectomy. Another reason is that traditional chemotherapy and radiotherapy are not effective for HCC. Because most of HCC patients are already in advanced stages at the time of diagnosis due to lack of sensitive and specific biomarkers in the clinic, together with high incidences of metastasis and recurrence, the prognosis of HCC is very poor. Therefore, there is an urgent need to identify new and effective molecular markers for early diagnosis and prognosis prediction and to find new molecular therapeutic targets for HCC patients.

In the past decades, many genes and cellular signaling pathways have been found to be implicated in the development and progression of HCC, including activation of mTOR (mammalian target of rapamycin) signaling pathway [3–5]. mTOR pathway has been shown to promote tumorigenesis via a coordinated phosphorylation of proteins important for cell growth, survival and metabolism, including EIF4EBP1 protein that regulates translation of mRNAs involved in oncogenic processes [6,7].

EIF4EBP1 (also known as 4EBP1) gene encodes a translation repressor protein that competitively binds to eukaryotic translation initiation factor 4E (EIF4E) to inhibit EIF4E complex assembly and thus the cap-dependent translation [8,9]. This protein is a major substrate of mTOR and acts as a crucial effector in mTOR signaling pathway. When mTORC1 is inhibited, hypophosphorylated EIF4EBP1 binds to and sequesters EIF4E [10]. In response to activated mTOR signaling pathway, EIF4EBP1 protein is phosphorylated, resulting in its dissociation from EIF4E and consequent initiation of mRNA translation, which promoters cell growth and proliferation [11].

Studies show that both EIF4EBP1 and EIF4E are involved in cancer development and progression. Up-regulated EIF4E plays an oncogenic role in carcinogenesis [12]. In contrast to EIF4E, EIF3A has been reported to be upregulated in various cancers but plays different roles in carcinogenesis: acts as tumor suppressor in squamous cell carcinomas and is correlated with better survival [13–15], and acts as tumor promoter in other epithelial carcinomas [16,17]. Altogether, of the 30 eukaryotic translation initiation factors (EIFs), some function as oncogene while some as tumor suppressor [18].

Similarly, EIF4EBP1, as the repressor of EIF4E, also may act as tumor promoter or tumor inhibitor. In general, phosphorylated EIF4EBP1 is considered as an indicator of oncogenic activity in tumor and associated with poor survival of cancer patients [19–22], while unphosphorylated EIF4EBP1 functions as a tumor suppressor [23–25]. However, the genomic locus for encoding EIF4EBP1 is amplificated, which results in overexpression of its corresponding mRNA in breast cancer, and the overexpressed mRNA is associated with poor prognosis of the patients [26]. Subsequently, total EIF4EBP1 protein was also reported to correlate with worse survival of cancer patients [22]. Therefore, EIF4EBP1 protein may not only act as a tumor suppressor but also as an oncogene. However, although many studies on EIF4EBP1 were conducted in human hepatoma cell lines [27–30], EIF4EBP1 expression level in human HCC tissues and its clinical significance have not been reported. In this study, we studied the expression of EIF4EBP1 mRNA and protein in HCC samples and evaluated its relationship with clinical features and prognosis of HCC patients.

## Materials and Methods

### Patients and tissue samples

All of the HCC patients underwent curative hepatectomy between June, 2004 and December, 2007, in Sun Yat-Sen University Cancer Center. Written informed consent was obtained from all of the patients before undergoing hepatectomy, in which patients agreed to use their medical record data and tissue samples for the research. Forty fresh paired tumors and adjacent noncancerous liver (NCL) tissues not less than 2 cm away from the HCC were immediately frozen in liquid nitrogen and stored at -80^°^C after resection. Another 88 archival formalin-fixed paraffin-embedded (FFPE) HCC tissues were obtained from the Department of Pathology of this cancer center. All of HCC samples were confirmed pathologically. The 88 patients also signed the written consent form when they underwent surgery. The patients didn’t receive any treatment before surgery, including chemotherapy and radiotherapy. Of the 88 HCC patients, 71 were men (80.68%) and 17 were women (19.32%), with a median age of 46 years (ranged from 13 to 72 years). The follow-up time ranged from 2 to 106 months with a median of 45.3 months. The characteristics of the 88 patients are listed in Table 1 and S1 Table in Supporting Information. This study was reviewed and approved by the Ethical Committee of Sun Yat-Sen University Cancer Center (Approval Number: GZR2013–077).

**Table 1:** Correlations between EIF4EBP1 expression and clinicopathological features in patients with HCC.

Parameter	4EBP1expression		*P* value
	Low N (%)	High N (%)	
Age			0.783
≥ 50y	16 (39.0)	17 (36.2)	
< 50y	25 (61.0)	30 (63.8)	
Gender			0.26
Female	10 (24.4)	7 (14.9)	
Male	31 (75.6)	40 (85.1)	
Serum HBsAg ^a^			0.242
Positive	33 (80.5)	42 (89.4)	
Negative	8 (19.5)	5 (10.6)	
Serum AFP ^b^			**0.003**
Positive	22 (53.7)	39 (83.0)	
Negative	19 (46.3)	8 (17.0)	
Liver Cirrhosis			0.695
Yes	29 (70.7)	35 (74.5)	
No	12 (29.3)	12 (25.5)	
Pathological Grade			0.085
I—II	25 (61.0)	20 (42.6)	
III—IV	16 (39.0)	27 (57.4)	
Tumor size (cm)			0.521
≥ 5	11 (36.7)	30 (51.7)	
< 5	19 (63.3)	28 (48.3)	
Tumor number			0.084
Single	33 (80.5)	30 (63.8)	
Multiple	8 (19.5)	17 (36.2)	
Tumor embolus			0.084
Yes	8 (19.5)	17 (36.2)	
No	33 (80.5)	30 (63.8)	
Tumor capsule			0.073
Yes	36 (87.8)	34 (72.3)	
No	5 (12.2)	13 (27.7)	
Postoperative Metastasis			0.467
Yes	4 (9.8)	7 (14.9)	
No	37 (90.2)	40 (85.1)	
Recurrence			0.373
Yes	12 (29.3)	18 (38.3)	
No	29 (70.7)	29 (61.7)	

^a^ HBsAg, hepatitis B surface antigen.

^b^ AFP, α-fetoprotein.

### RNA extraction and quantitative real-time PCR

Total RNA was extracted from frozen fresh tissues using TRIzol reagent (Invitrogen) according to the manufacturer’s instruction. Then 2 μg of total RNA were used for producing cDNA using M-MLV reverse transcriptase following the protocol provided by the manufacture (Promega, USA). Real-time PCR was performed in triplicate for each sample using an Applied Biosystems PRISM 7900HT system (Foster City, CA, USA) with a 15 μl reaction volume containing 0.5 μl cDNA, 7.5 μl of SYBR Green master mix (Invitrogen, USA) and 200 nM of each forward primer and reverse primer. GAPDH was detected by RT-PCR as an internal control. Primer sequences for EIF4EBP1 and GAPDH were as follows: EIF4EBP1, forward: CCCGCTTATCTTCTGGGCTA and reverse: CTATGACCGGAAATTCCTGATGG;; GAPDH, forward: CTCCTCCTGTTCGACAGTCAGC and reverse: CCCA ATACGACCAAATCCGTT. The real-time PCR cycle was as follows: 95°C for 10 minutes, 40 cycles of 95°C for 30 s and 60°C for 60 s, and a dissociation stage.

### Western blot

Of the 40 paired HCC samples, randomly selected 8 HCCs and matched NCL tissues were homogenized in a RIPA lysis buffer. After centrifugation at 12,000 rpm and 4°C for 20 minutes, approximate 40 μg of protein were run on a 12% SDS-PAGE gel and transferred to polyvinylidene difluoride membrane (PVDF, Millipore). After blocking non-specific binding sites for 60 minutes with 5% non-fat milk, membranes were incubated with rabbit monoclonal antibody against EIF4EBP1 (1:1000; Cell signaling Technology), and GAPDH (1:1000; Sigma-Aldrich) at 4°C over night, respectively. Then membranes were washed three times with TBST for 15 minutes each time and incubated with HRP-conjugated anti-rabbit secondary antibody (1:1000; Santa Cruz) for 45 minutes at room temperature. The membrane was developed by an enhanced chemiluminescence system (ECL; cell signaling) after washed three times with TBST. The intensity of the protein bands was determined by using Image J software (http://rsb.info.nih.gov/).

### Immunohistochemical analysis

All formalin-fixed paraffin-embedded HCC specimens were sectioned (4 μm) and baked at 65°C for 2 hours, and then deparaffinized with xylene and rehydrated in decreasing concentrations of alcohol. For antigen retrieval, the sections were boiled in EDTA solution (1 mmol/L, pH 8.0) for 20 minutes in a microwave oven and then cooled to room temperature. The endogenous peroxidase was inactivated with 0.3% hydrogen peroxide for 10 minutes. After being rinsed three times in phosphate buffer saline (PBS), the sections were incubated with anti-EIF4EBP1 antibody (Cell signaling Technology) diluted in a working solution (1:100) at 4°C overnight, and then incubated with horseradish peroxidase-conjugated secondary antibody (Envision Detection Kit, GK500705, Gene Tech) at 37°C for 30 minutes. Finally, the visualization signal was developed with 3, 3’-diaminobenzidine tetrahydrochloeide (DAB) and then all of the slides were counterstained with hematoxylin, dehydrated in increasing concentrations of alcohol and cover-slipped with neutral permount. As a negative control, the primary antibody was replaced by PBS under the same experimental conditions.

The expression levels of EIF4EBP1 protein in samples were quantified with semi-quantitative immunostaining score (ISS) method. Two pathologists blinded to the clinical data scored the EIF4EBP1 staining independently. The intensity of immunostaining was defined as 0–3 (0, negative; 1, weak; 2, moderate; 3, strong), and the percentage of positive immunostaining was scored as 0 to 100%. The ISS is calculated for each sample by the multiplication of the intensity score by the percentage score, which results in an ISS between 0 (no staining) and 300 (maximum staining). Based on the ISS, patients were divided into high- and low-expression groups by the median score.

### Statistical analysis

To compare the relative expression levels of EIF4EBP1, the 2^-ΔΔCt^ was used to represent the relative expression level of mRNA in each sample normalized by GAPDH. Paired Student’s t test was performed to analyze the difference of mRNA expression levels between HCC and NCL tissues. If the data did not meet the normal distribution, Mann–Whitney U test would be used. The quantitative values of qRT-PCR were expressed as mean ± SD.

The χ^2^ test or Fisher’s exact test and Mann-Whitney U test were used to analyze the correlations between EIF4EBP1 protein expression level and clinicopathological features. We used the Kaplan Meier method and the log-rank test to estimate overall survival (OS) and disease-free survival (DFS). The prognostic significance of each clinicopathologic characteristic was determined using univariate Cox regression analysis. Parameters that were significantly related to survival rate in the univariate analysis were entered into the multivariate analysis. We did a multivariate Cox regression analysis using a backward stepwise approach to test if EIF4EBP1 was an independent prognostic factor of OS and DFS. The significance was defined as *P* value less than 0.05. All statistical analyses were performed using SPSS16.0 (SPSS Inc, Chicago, IL) and/or Graph Pad Prism 5 software (www.graphpad.com).

## Results

### EIF4EBP1 mRNA level is up-regulated in HCC

**Fig 1:**
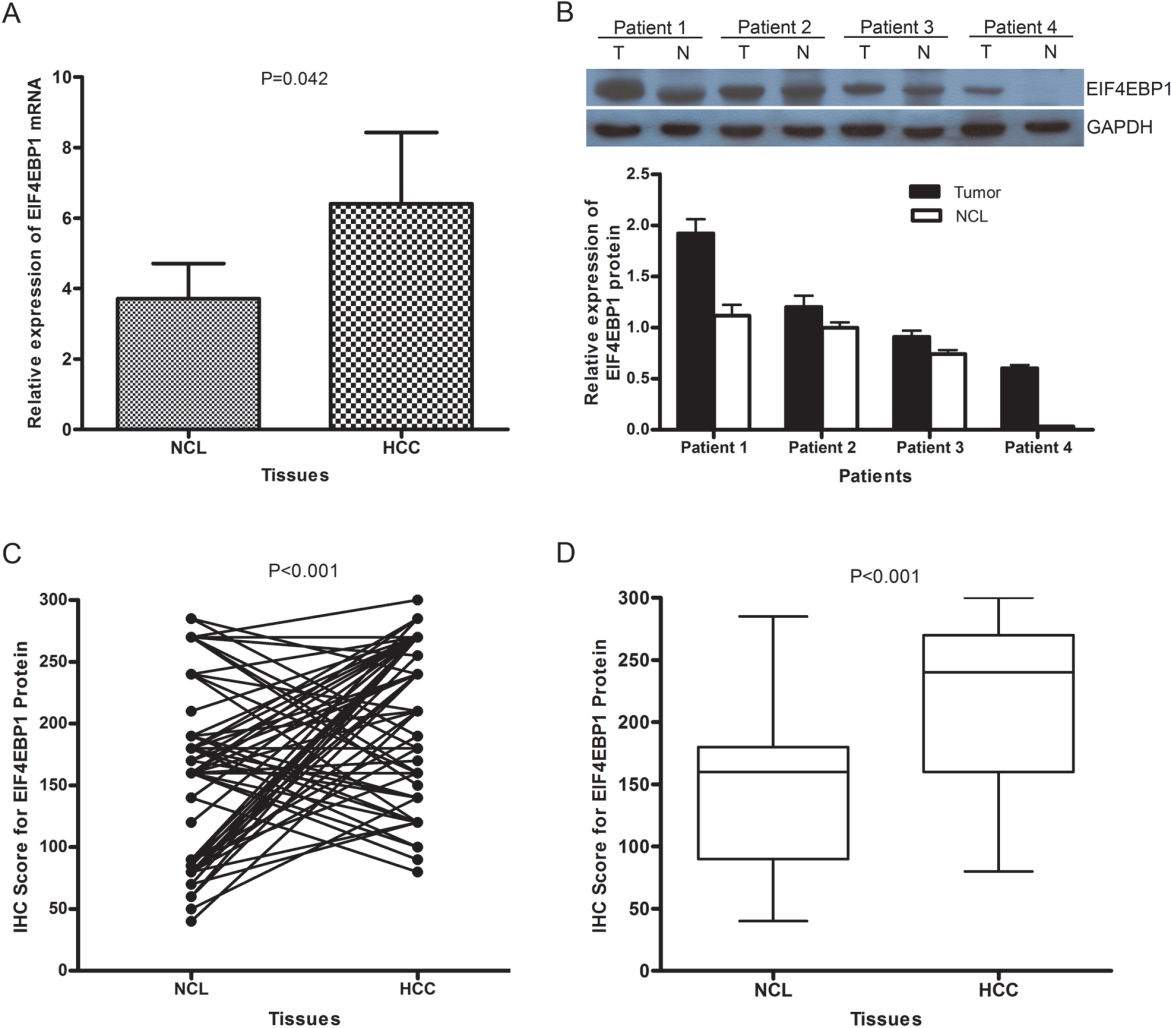
The expression levels of both EIF4EBP1 mRNA and protein in HCCs are significantly higher than in the matched adjacent NCLs. **(A)** The relative EIF4EBP1 mRNA level was examined by RT-PCR. Shown is the relative expression level of EIF4EBP1 mRNA in HCC tissues compared with the matched NCLs (mean±SD; n = 40, Paired *t* test, *P* = 0.042); **(B)** EIF4EBP1 protein expression levels in 8 HCC tissues and the matched NCLs were detected by Western blot (representative image for 4 pairs of HCC samples), and lower panel shows the relative expression levels of EIF4EBP1 protein. **(C)** The relative expression level in each HCC tissue by immunohistochemistry is compared with that in its corresponding NCL tissue; **(D)** Relative expression level of EIF4EBP1 protein in 88 HCC tissues and the matched adjacent NCL tissues as measured by immunohistochemistry. (Y axis indicates the immunostaining score).

The EIF4EBP1 mRNA level was reported to be up-regulated in some cancer types. However, the mRNA level in HCC has not been explored. To investigate the expression level of EIF4EBP1 mRNA in patients with HCC, we carried out the qRT-PCR assay in 40 paired HCC and NCL tissues. The result demonstrate higher expression of EIF4EBP1 mRNA in 60% (24/40) of HCC tissues compared with NCL tissues, and this increase is statistically significant (*P* = 0.044,Fig. 1A). This result is consistent with previously reported results in other cancers [22,26].

### The EIF4EBP1 protein is overexpression in HCC tissues

In general, higher mRNA level will lead to higher level of its encoded protein. To confirm the protein level, Western blot analysis was employed to detect the expression of EIF4EBP1 protein in 8 paired HCC and noncancerous liver tissues. As expected, the result also shows a high expression level of EIF4EBP1 protein in HCC tissues compared with the paired noncancerous liver tissues (Fig. 1B), which is consistent with the result of qRT-PCR.

**Fig 2:**
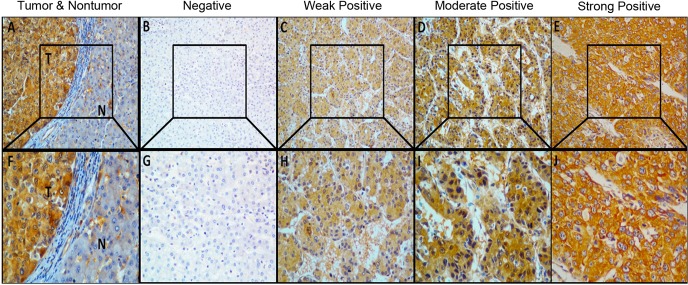
Immunohistochemical staining of EIF4EBP1 protein in HCC and NCL. Eighty-eight archival formalin-fixed paraffin-embedded paired HCC samples were analyzed by immunohistochemistry with EIF4EBP1-specific antibody. Shown are representative results. The lower panel shows magnified images of the upper panel. **(A)** and **(F)**: Immunostaining of HCC tumor area and the adjacent non-tumor area. **(B)** and **(G)**: Noncancerous liver tissue was scored as no staining for EIF4EBP1 (Score 0). **(C)** and **(H)**: Well-differentiated HCC was scored as weak staining for EIF4EBP1 (Score 1). **(D)** and **(I)**: Moderately differentiated HCC was scored as moderate staining for EIF4EBP1 (Score 2). **(E)** and **(J)**: Poorly differentiated HCC was scored as strong staining for EIF4EBP1 (Score 3). Note: N: Noncancerous liver tissue; T: Tumor tissue; A–E with x200 magnification; F–J with x400 magnification.

To explore the clinical significance of overexpressed EIF4EBP1 protein in HCC patients, another 88 archival formalin-fixed paraffin-embedded HCC samples were detected by immunohistochemical staining. The result shows that 53% (47/88) of HCCs exhibit high expression of EIF4EBP1 compared with the matched NCLs (Fig. 1C, and S1 Table in Supporting Information) and that EIF4EBP1 expression level in HCC is significantly higher than that in NCL (Fig. 1D), which is similar to the percentage of high EIF4EBP1 mRNA expression in 40 HCCs, confirming EIF4EBP1 overexpression in HCC. EIF4EBP1 staining signal is mainly present in the cytoplasm (Fig. 2) and also in the nuclei of HCC cells (Fig. 2D, 2I). We observed prominent contrast of positive staining in tumor areas and negative staining in the adjacent non-tumor tissues (Fig. 2A, 2F). In addition, in a number of HCC samples, well-differentiated HCCs present or weak positive staining (Fig. 2C, 2H), moderately differentiated ones display medium positive staining (Fig. 2D, 2I), and poorly differentiated ones show highly positive staining for EIF4EBP1 (Fig. 2E, 2J). Overall, EIF4EBP1 protein is significantly up-regulated in HCC tissues compared with the paired NCL tissues (*P* < 0.001, Fig. 1B).

### EIF4EBP1 protein overexpression is associated with disease progression of HCC patients

To understand the clinical significance of EIF4EBP1 expression in HCC, patients first were divided into a high- or low-expression group based on their immunostaining scores of HCC tissue with the median staining score (210) as the cutoff. The relationship between the protein level and the clinicopathologic parameters was analyzed. Table 1 shows the correlations between EIF4EBP1 and clinicopathologic features. High EIF4EBP1 expression is significantly associated with serum AFP positive (*P* = 0.003), and marginally significantly associated with pathological grade (*P* = 0.085), tumor number (*P* = 0.084), tumor embolus (*P* = 0.084) and capsulation (*P* = 0.073), suggesting that EIF4EBP1 is involved in HCC progression.

### EIF4EBP1 protein is an independent predictor for survival in HCC patients

**Fig 3:**
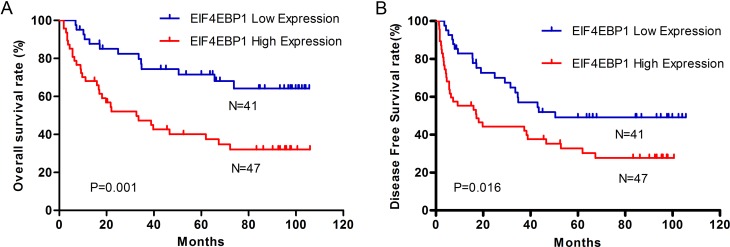
Kaplan–Meier survival curves of overall survival and disease-free survival in HCC patients with high- and low-expression of EIF4EBP1 protein. HCC patients with high expression of EIF4EBP1 have significantly lower overall survival rate **(A)** (log-rank, *P* = 0.0013) and disease-free survival rate **(B)** (log-rank, *P* = 0.0156) than those with low expression of EIF4EBP1.

To evaluate the prognostic value of EIF4EBP1 protein in HCC patients, survival curves were evaluated by Kaplan–Meier method and compared by the log-rank test. Patients with high expression of EIF4EBP1 have a significantly poorer 5-year overall survival (40.3% vs 73.6%, Fig. 3A) and 5-year disease-free survival (33.0% vs 49.0%, Fig. 3B) than those with low expression. Univariate analysis was carried out using Cox proportional hazard model to evaluate the impact of EIF4EBP1 expression and clinicopathological features on OS and DFS in HCC patients. The results indicate that EIF4EBP1 expression, pathology grade, tumor number, encapsulation,embolus and recurrence are significantly associated with OS and DFS (Table 2). Multivariate Cox regression analysis demonstrate that EIF4EBP1 expression is an independent predictor of OS (HR, 2.285; 95% CI, 1.154–4.527; *P* = 0.018,Table 2) and DFS (HR, 1.901; 95% CI, 1.067–3.386; *P* = 0.029,Table 2), and other independent predictors for survival included tumor number, embolus and recurrence.

**Table 2:** Univariate and multivariate analysis of overall survival in patients with HCC.

Variables	Univariate analysis			Multivariate analysis		
	HR	95% CI	*P* Value	HR	95% CI	*P* Value
**OVERALL SURVIVAL**						
4EBP1 Expression	2.797	1.456–5.372	**0.002**	2.285	1.154–4.527	**0.018**
Age (≥50y vs <50y)	0.978	0.531–1.803	0.943			
Gender (Male vs Female)	0.909	0.421–1.963	0.808			
Serum HBsAg (positive vs negative)	0.756	0.336–1.703	0.500			
Serum AFP (positive vs negative)	1.905	0.937–3.872	0.075			
Cirrhosis (yes vs no)	1.067	0.548–2.078	0.849			
Pathology Grade (I—II vs III—IV)	2.004	1.086–3.697	**0.026**			
Tumor Size (≥ 5 cm vs 5 cm)	1.823	0.993–3.348	0.053			
Tumor Number (single vs multiple)	4.130	2.235–7.629	**<0.001**	3.613	1.916–6.812	**<0.001**
Tumor Embolus (yes vs no)	2.850	1.546–5.252	**0.001**			
Tumor Encapsulation (yes vs no)	0.373	0.193–0.724	**0.004**	0.445	0.224–0.884	**0.021**
Postoperative Metastasis (yes vs no)	1.485	0.660–3.343	0.340			
Postoperative Recurrence (yes vs no)	2.277	1.246–4.162	0.007	2.294	1.237–4.252	0.008
**DISEASE-FREE SURVIVAL**						
4EBP1 Expression	1.960	1.123–3.418	**0.018**	1.901	1.067–3.386	**0.029**
Age (≥50y vs <50y)	1.011	0.583–1.755	0.968			
Gender (M vs F)	0.861	0.431–1.720	0.672			
Serum HBsAg (positive vs negative)	1.068	0.481–2.367	0.872			
Serum AFP (positive vs negative)	2.176	1.142–4.148	**0.018**			
Cirrhosis (yes vs no)	1.225	0.665–2.258	0.514			
Pathology Grade (I—II vs III—IV)	1.275	0.743–2.188	0.377			
Tumor Size (≥ 5 cm vs < 5 cm)	2.342	1.139–4.814	**0.021**			
Tumor Number (single vs multiple)	3.015	1.738–5.231	**<0.001**	4.687	2.566–8.561	**<0.001**
Tumor Embolus (yes vs no)	2.755	1.579–4.806	**<0.001**	1.959	1.112–3.451	**0.020**
Tumor Capsule (yes vs no)	0.503	0.271–0.934	**0.029**			
Postoperative Metastasis (yes vs no)	1.571	0.740–3.336	0.24			
Postoperative Recurrence (yes vs no)	6.948	3.866–12.486	**<0.001**	9.555	5.109–17.871	**<0.001**

## Discussion

Up till now, the clinical significance of EIF4EBP1 expression in human HCC has not been studied. Therefore, we hypothesized that EIF4EBP1 plays an important role in development and progression of HCC. To test the hypothesis, we examined the expression of EIF4EBP1 at mRNA and protein levels in the paired HCC and NCL samples using qRT-PCR, Western blot and immunohistochemistry and then evaluated the clinical significance of its protein expression. As expected, EIF4EBP1 mRNA and protein are up-regulated in most of HCC cancer tissues compared with their corresponding NCL tissues. In immunohistochemical analysis, EIF4EBP1 protein is not only present in cytoplasm but also in the nucleus, which was in line with a previous report by Grzmil et al [31]. EIF4EBP1 protein located in the nucleus other than cytoplasm indicates that EIF4EBP1 protein may be regulated by subcellular localization or has functions in addition to repression of eIF4E during carcinogenesis. Furthermore, overexpression of EIF4EBP1 protein in HCC is significantly or marginally significantly associated with important clinical features. The relatively small sample size limits the significance for the relationship of EIF4EBP1 expression with certain important clinical features, such as pathological grade, tumor number and embolus. If the sample size is large enough, EIF4EBP1 expression may prove to be significantly associated with these clinical features. Together, our data suggest that EIF4EBP1 protein may play a key role during the development and progression of HCC. More importantly, Kaplan-Meier survival analysis reveals that the high EIF4EBP1 protein expression is significantly correlated to a poor prognosis of HCC patients after surgical resection. Multivariate Cox analysis demonstrates that EIF4EBP1 protein is an independent prognostic factor for OS and DFS. Consistent with our observations, EIF4EBP1 mRNA is up-regulated in 61.8% HCCs (see the Dataset GSE4108 in NCBI Gene Expression Ominibus [www.ncbi.nlm.nih.gov/geo/query/acc.cgi?acc=GSE4108]). Altogether, our findings suggest that EIF4EBP1 functions as an oncogene in HCC and may serve as a potential new molecular marker for predicting the prognosis in HCC patients after surgical resection.

EIF4EBP1 is commonly regarded as a tumor suppressor because of its role in repressing translational initiation of mRNAs associated with oncogenic transformation [23]. As mentioned above, however, Karlsson and his colleagues demonstrated that EIF4EBP1 mRNA and total protein were associated with progression and poor prognosis in breast cancer [22, 26], which is in agreement with our results in this study. The evidence provided by our study and others indicates that EIF4EBP1 potentially functions as an oncogene in some cancer types. There may be two reasons for non-phosphorylated EIF4EBP1 protein functioned as an oncogene in our study. One is that non-phosphorylated EIF4EBP1 protein is localized in nucleus, which plays a different role than that in cytoplasm; the other one is that more non-phosphorylated EIF4EBP1 protein may result in more phosphorylated EIF4EBP1 because total EIF4BP1 protein level is positively correlated with phosphorylated EIF4EBP1 protein level [22]. We also detected both EIF4EBP1 and phosphorylated EIF4EBP1 in 26 of 88 HCC samples by immunohistochemistry (data not show here), and the results show that EIF4EBP1 level also is positively correlated with phosphorylated EIF4EBP1 level (r = 0.498, *P* = 0.01). On the basis of the oncogenic role of EIF4EBP1 in HCC development and progression, it is possible to target both EIF4EBP1 and phosphorylated EIF4EBP1 proteins for HCC patients with EIF4EBP1 overexpression.

In conclusion, this study demonstrates for the first time that EIF4EBP1 expression is up-regulated in HCC tissues at both mRNA and protein levels, and high expression of EIF4EBP1 protein indicates poor prognosis of HCC patients. Our findings suggest that EIF4EBP1 is a novel prognostic biomarker for HCC and may serve as a potential therapeutic target for HCC.

## Supporting Information

S1 Table Immunostaining score and clinical features of 88 HCC cases. (DOCX)

## References

[1] Ferlay J, Shin HR, Bray F, Forman D, Mathers C, et al (2010) Estimates of worldwide burden of cancer in 2008: GLOBOCAN 2008. Int J Cancer 127: 2893–2917. 10.1002/ijc.25516 21351269

[2] Jemal A, Bray F, Center MM, Ferlay J, Ward E, et al (2011) Global cancer statistics. CA Cancer J Clin 61: 69–90. 10.3322/caac.20107 21296855

[3] Villanueva A, Chiang DY, Newell P, Peix J, Thung S, et al (2008) Pivotal role of mTOR signaling in hepatocellular carcinoma. Gastroenterology 135: 1972–1983, 1983 e1971–1911. 10.1053/j.gastro.2008.08.008 18929564 PMC2678688

[4] Zender L, Villanueva A, Tovar V, Sia D, Chiang DY, et al (2010) Cancer gene discovery in hepatocellular carcinoma. J Hepatol 52: 921–929. 10.1016/j.jhep.2009.12.034 20385424 PMC2905725

[5] Wang C, Cigliano A, Delogu S, Armbruster J, Dombrowski F, et al (2013) Functional crosstalk between AKT/mTOR and Ras/MAPK pathways in hepatocarcinogenesis: implications for the treatment of human liver cancer. Cell Cycle 12: 1999–2010. 10.4161/cc.25099 23759595 PMC3737302

[6] Magnuson B, Ekim B, Fingar DC (2012) Regulation and function of ribosomal protein S6 kinase (S6K) within mTOR signalling networks. Biochem J 441: 1–21. 10.1042/BJ20110892 22168436

[7] Li X, Li Z, Zhou W, Xing X, Huang L, et al (2013) Overexpression of 4EBP1, p70S6K, Akt1 or Akt2 differentially promotes Coxsackievirus B3-induced apoptosis in HeLa cells. Cell Death Dis 4: e803–809. 10.1038/cddis.2013.331 24030155 PMC3789189

[8] Harris TE, Lawrence JC Jr. (2003) TOR signaling. Sci STKE 2003: re15 14668532 10.1126/stke.2122003re15

[9] Gingras AC, Raught B, Sonenberg N (1999) eIF4 initiation factors: effectors of mRNA recruitment to ribosomes and regulators of translation. Annu Rev Biochem 68: 913–963. 10872469 10.1146/annurev.biochem.68.1.913

[10] Aoyagi M, Gaspar M, Shenk TE (2010) Human cytomegalovirus UL69 protein facilitates translation by associating with the mRNA cap-binding complex and excluding 4EBP1. Proc Natl Acad Sci U S A 107: 2640–2645. 10.1073/pnas.0914856107 20133758 PMC2823912

[11] Thoreen CC (2013) Many roads from mTOR to eIF4F. Biochem Soc Trans 41: 913–916. 10.1042/BST20130082 23863155

[12] Heikkinen T, Korpela T, Fagerholm R, Khan S, Aittomaki K, et al (2013) Eukaryotic translation initiation factor 4E (eIF4E) expression is associated with breast cancer tumor phenotype and predicts survival after anthracycline chemotherapy treatment. Breast Cancer Res Treat 141: 79–88. 10.1007/s10549-013-2671-2 23974830

[13] Dellas A, Torhorst J, Bachmann F, Banziger R, Schultheiss E, et al (1998) Expression of p150 in cervical neoplasia and its potential value in predicting survival. Cancer 83: 1376–1383. 9762939 10.1002/(sici)1097-0142(19981001)83:7<1376::aid-cncr15>3.0.co;2-1

[14] Chen G, Burger MM (1999) p150 expression and its prognostic value in squamous-cell carcinoma of the esophagus. Int J Cancer 84: 95–100. 10096238 10.1002/(sici)1097-0215(19990420)84:2<95::aid-ijc1>3.0.co;2-n

[15] Spilka R, Laimer K, Bachmann F, Spizzo G, Vogetseder A, et al (2012) Overexpression of eIF3a in Squamous Cell Carcinoma of the Oral Cavity and Its Putative Relation to Chemotherapy Response. J Oncol 2012: 901956 10.1155/2012/901956 22619676 PMC3347757

[16] Haybaeck J, O'Connor T, Spilka R, Spizzo G, Ensinger C, et al (2010) Overexpression of p150, a part of the large subunit of the eukaryotic translation initiation factor 3, in colon cancer. Anticancer Res 30: 1047–1055. 20530408

[17] Spilka R, Ernst C, Bergler H, Rainer J, Flechsig S, et al (2014) eIF3a is over-expressed in urinary bladder cancer and influences its phenotype independent of translation initiation. Cell Oncol (Dordr) 37: 253–267. 10.1007/s13402-014-0181-9 25070653 PMC13004439

[18] Spilka R, Ernst C, Mehta AK, Haybaeck J (2013) Eukaryotic translation initiation factors in cancer development and progression. Cancer Lett 340: 9–21. 10.1016/j.canlet.2013.06.019 23830805

[19] No JH, Jeon YT, Park IA, Kim YB, Kim JW, et al (2011) Activation of mTOR signaling pathway associated with adverse prognostic factors of epithelial ovarian cancer. Gynecol Oncol 121: 8–12. 10.1016/j.ygyno.2010.12.364 21276607

[20] Castellvi J, Garcia A, Rojo F, Ruiz-Marcellan C, Gil A, et al (2006) Phosphorylated 4E binding protein 1: a hallmark of cell signaling that correlates with survival in ovarian cancer. Cancer 107: 1801–1811. 16983702 10.1002/cncr.22195

[21] Rojo F, Najera L, Lirola J, Jimenez J, Guzman M, et al (2007) 4E-binding protein 1, a cell signaling hallmark in breast cancer that correlates with pathologic grade and prognosis. Clin Cancer Res 13: 81–89. 17200342 10.1158/1078-0432.CCR-06-1560

[22] Karlsson E, Perez-Tenorio G, Amin R, Bostner J, Skoog L, et al (2013) The mTOR effectors 4EBP1 and S6K2 are frequently coexpressed, and associated with a poor prognosis and endocrine resistance in breast cancer: a retrospective study including patients from the randomised Stockholm tamoxifen trials. Breast Cancer Res 15: R96 24131622 10.1186/bcr3557 PMC3978839

[23] Armengol G, Rojo F, Castellvi J, Iglesias C, Cuatrecasas M, et al (2007) 4E-binding protein 1: a key molecular "funnel factor" in human cancer with clinical implications. Cancer Res 67: 7551–7555. 17699757 10.1158/0008-5472.CAN-07-0881

[24] Martineau Y, Azar R, Bousquet C, Pyronnet S (2013) Anti-oncogenic potential of the eIF4E-binding proteins. Oncogene 32: 671–677. 10.1038/onc.2012.116 22508483

[25] Cai W, Ye Q, She QB (2014) Loss of 4E-BP1 function induces EMT and promotes cancer cell migration and invasion via cap-dependent translational activation of snail. Oncotarget 5: 6015–6027. 24970798 10.18632/oncotarget.2109 PMC4171609

[26] Karlsson E, Waltersson MA, Bostner J, Perez-Tenorio G, Olsson B, et al (2011) High-resolution genomic analysis of the 11q13 amplicon in breast cancers identifies synergy with 8p12 amplification, involving the mTOR targets S6K2 and 4EBP1. Genes Chromosomes Cancer 50: 775–787. 10.1002/gcc.20900 21748818

[27] Li M, Wang J, Yang L, Gao P, Tian QB, et al (2014) eRF3b, a biomarker for hepatocellular carcinoma, influences cell cycle and phosphoralation status of 4E-BP1. PLoS One 9: e86371 10.1371/journal.pone.0086371 24466059 PMC3900531

[28] Weng Q, Zhang J, Cao J, Xia Q, Wang D, et al (2011) Q39, a quinoxaline 1,4-Di-N-oxide derivative, inhibits hypoxia-inducible factor-1alpha expression and the Akt/mTOR/4E-BP1 signaling pathway in human hepatoma cells. Invest New Drugs 29: 1177–1187. 10.1007/s10637-010-9462-y 20524035

[29] Garcia-Maceira P, Mateo J (2009) Silibinin inhibits hypoxia-inducible factor-1alpha and mTOR/p70S6K/4E-BP1 signalling pathway in human cervical and hepatoma cancer cells: implications for anticancer therapy. Oncogene 28: 313–324. 10.1038/onc.2008.398 18978810

[30] Scheper GC, Mulder J, Kleijn M, Voorma HO, Thomas AA, et al (1997) Inactivation of eIF2B and phosphorylation of PHAS-I in heat-shocked rat hepatoma cells. J Biol Chem 272: 26850–26856. 9341116 10.1074/jbc.272.43.26850

[31] Grzmil M, Huber RM, Hess D, Frank S, Hynx D, et al (2014) MNK1 pathway activity maintains protein synthesis in rapalog-treated gliomas. J Clin Invest 124: 742–754. 10.1172/JCI70198 24401275 PMC3904612

