# Supplementary material for: EIF4EBP1 Overexpression Is Associated with Poor Survival and Disease Progression in Patients with Hepatocellular Carcinoma
Source: PLoS One. 2015 Feb 6;10(2):e0117493. doi: 10.1371/journal.pone.0117493 (PMC4319970; doi:10.1371/journal.pone.0117493)
Supplement: S1 Table — (DOCX) [file pone.0117493.s001.docx]

Supporting information

Table S1. Immunostaining score and clinical features of 88 HCC cases

| Case # | 4EBP1 Score | Gende | | Age | | HBsAg | | AFP | | Cirrh | | T grade | | T size | | T # | Embolus | | capsule | | relapse | | metast | |
| --- | --- | --- | --- | --- | --- | --- | --- | --- | --- | --- | --- | --- | --- | --- | --- | --- | --- | --- | --- | --- | --- | --- | --- | --- |
| Case 1 | 180 | 1 | 0 | | 1 | | 1 | | 1 | | 1 | | 0 | | 0 | | | 0 | 1 | 0 | | 0 | |  |
| Case 2 | 140 | 1 | 1 | | 1 | | 0 | | 1 | | 1 | | 0 | | 0 | | | 1 | 1 | 0 | | 0 | |  |
| Case 3 | 140 | 1 | 1 | | 0 | | 0 | | 0 | | 1 | | 1 | | 0 | | | 0 | 0 | 0 | | 0 | |  |
| Case 4 | 140 | 1 | 0 | | 1 | | 0 | | 1 | | 0 | | 1 | | 0 | | | 0 | 1 | 0 | | 0 | |  |
| Case 5 | 270 | 0 | 0 | | 1 | | 1 | | 0 | | 1 | | 1 | | 1 | | | 1 | 0 | 0 | | 0 | |  |
| Case 6 | 30 | 1 | 0 | | 1 | | 1 | | 1 | | 0 | | 1 | | 0 | | | 0 | 1 | 1 | | 0 | |  |
| Case 7 | 270 | 1 | 1 | | 1 | | 1 | | 1 | | 1 | | 1 | | 1 | | | 1 | 1 | 1 | | 0 | |  |
| Case 8 | 120 | 0 | 1 | | 0 | | 1 | | 1 | | 1 | | 1 | | 0 | | | 1 | 1 | 0 | | 0 | |  |
| Case 9 | 270 | 0 | 0 | | 0 | | 1 | | 0 | | 1 | | 1 | | 0 | | | 1 | 1 | 0 | | 0 | |  |
| Case 10 | 210 | 1 | 0 | | 1 | | 0 | | 1 | | 1 | | 1 | | 0 | | | 0 | 1 | 0 | | 0 | |  |
| Case 11 | 170 | 1 | 0 | | 1 | | 1 | | 0 | | 1 | | 1 | | 0 | | | 1 | 1 | 0 | | 0 | |  |
| Case 12 | 270 | 1 | 1 | | 1 | | 1 | | 1 | | 0 | | 1 | | 0 | | | 1 | 1 | 1 | | 0 | |  |
| Case 13 | 270 | 1 | 0 | | 1 | | 1 | | 0 | | 1 | | 1 | | 0 | | | 1 | 1 | 1 | | 0 | |  |
| Case 14 | 190 | 1 | 1 | | 0 | | 0 | | 1 | | 0 | | 0 | | 0 | | | 0 | 1 | 0 | | 0 | |  |
| Case 15 | 160 | 1 | 0 | | 1 | | 0 | | 0 | | 0 | | 1 | | 1 | | | 0 | 0 | 0 | | 0 | |  |
| Case 16 | 270 | 1 | 1 | | 1 | | 1 | | 1 | | 0 | | 0 | | 0 | | | 0 | 1 | 0 | | 0 | |  |
| Case 17 | 80 | 1 | 0 | | 1 | | 1 | | 0 | | 0 | | 1 | | 0 | | | 0 | 1 | 1 | | 0 | |  |
| Case 18 | 240 | 1 | 1 | | 1 | | 0 | | 1 | | 1 | | 0 | | 0 | | | 0 | 1 | 0 | | 0 | |  |
| Case 19 | 180 | 1 | 0 | | 1 | | 0 | | 1 | | 1 | | 0 | | 0 | | | 0 | 1 | 0 | | 0 | |  |
| Case 20 | 270 | 1 | 0 | | 1 | | 1 | | 0 | | 0 | | 1 | | 1 | | | 0 | 1 | 0 | | 0 | |  |
| Case 21 | 270 | 1 | 0 | | 1 | | 1 | | 1 | | 0 | | 0 | | 1 | | | 0 | 1 | 1 | | 0 | |  |
| Case 22 | 270 | 0 | 0 | | 1 | | 1 | | 1 | | 0 | | 1 | | 1 | | | 0 | 0 | 0 | | 0 | |  |
| Case 23 | 140 | 1 | 1 | | 1 | | 0 | | 1 | | 0 | | 0 | | 0 | | | 0 | 1 | 1 | | 0 | |  |
| Case 24 | 285 | 1 | 1 | | 1 | | 1 | | 1 | | 1 | | 1 | | 1 | | | 0 | 0 | 0 | | 0 | |  |
| Case 25 | 180 | 0 | 1 | | 0 | | 1 | | 0 | | 1 | | 1 | | 1 | | | 0 | 0 | 0 | | 0 | |  |
| Case 26 | 255 | 1 | 1 | | 1 | | 1 | | 1 | | 0 | | 1 | | 0 | | | 0 | 1 | 0 | | 0 | |  |
| Case 27 | 30 | 1 | 0 | | 1 | | 1 | | 1 | | 0 | | 1 | | 1 | | | 0 | 1 | 1 | | 0 | |  |
| Case 28 | 120 | 1 | 1 | | 1 | | 0 | | 1 | | 0 | | 0 | | 0 | | | 0 | 1 | 0 | | 0 | |  |
| Case 29 | 270 | 1 | 0 | | 1 | | 1 | | 1 | | 1 | | 1 | | 0 | | | 0 | 1 | 1 | | 0 | |  |
| Case 30 | 300 | 1 | 1 | | 0 | | 0 | | 0 | |  | | 1 | | 0 | | | 0 | 1 | 0 | | 0 | |  |
| Case 31 | 60 | 1 | 1 | | 1 | | 0 | | 0 | | 0 | | 1 | | 0 | | | 0 | 1 | 1 | | 0 | |  |
| Case 32 | 270 | 1 | 0 | | 1 | | 1 | | 1 | | 1 | | 1 | | 0 | | | 1 | 1 | 0 | | 0 | |  |
| Case 33 | 240 | 1 | 0 | | 1 | | 1 | | 1 | | 1 | | 0 | | 1 | | | 1 | 1 | 1 | | 0 | |  |
| Case 34 | 270 | 1 | 0 | | 1 | | 1 | | 1 | | 1 | | 1 | | 0 | | | 1 | 1 | 0 | | 1 | |  |
| Case 35 | 180 | 1 | 0 | | 1 | | 1 | | 1 | | 1 | | 1 | | 0 | | | 0 | 1 | 1 | | 0 | |  |
| Case 36 | 100 | 0 | 0 | | 1 | | 1 | | 1 | | 0 | | 0 | | 0 | | | 0 | 1 | 0 | | 0 | |  |
| Case 37 | 210 | 1 | 1 | | 1 | | 1 | | 0 | | 0 | | 1 | | 1 | | | 1 | 0 | 0 | | 1 | |  |
| Case 38 | 140 | 0 | 1 | | 0 | | 1 | | 1 | | 1 | | 1 | | 0 | | | 1 | 1 | 1 | | 0 | |  |
| Case 39 | 180 | 0 | 0 | | 1 | | 1 | | 0 | | 1 | | 0 | | 1 | | | 0 | 1 | 0 | | 0 | |  |
| Case 40 | 160 | 1 | 0 | | 1 | | 1 | | 1 | | 0 | | 0 | | 0 | | | 0 | 1 | 0 | | 0 | |  |
| Case 41 | 210 | 1 | 1 | | 1 | | 1 | | 1 | | 1 | | 0 | | 0 | | | 0 | 1 | 0 | | 0 | |  |
| Case 42 | 160 | 1 | 0 | | 1 | | 1 | | 1 | | 1 | | 1 | | 0 | | | 0 | 1 | 0 | | 0 | |  |
| Case 43 | 120 | 1 | 0 | | 1 | | 1 | | 1 | | 0 | | 1 | | 0 | | | 0 | 1 | 1 | | 0 | |  |
| Case 44 | 270 | 1 | 1 | | 1 | | 0 | | 0 | | 0 | | 1 | | 0 | | | 0 | 1 | 0 | | 0 | |  |
| Case 45 | 285 | 1 | 0 | | 1 | | 0 | | 1 | | 0 | | 1 | | 0 | | | 0 | 1 | 0 | | 0 | |  |
| Case 46 | 270 | 1 | 0 | | 1 | | 1 | | 0 | | 1 | | 0 | | 0 | | | 0 | 1 | 0 | | 0 | |  |
| Case 47 | 30 | 0 | 0 | | 1 | | 1 | | 0 | | 1 | | 1 | | 0 | | | 1 | 1 | 1 | | 1 | |  |
| Case 48 | 270 | 1 | 0 | | 1 | | 1 | | 1 | | 1 | | 1 | | 1 | | | 1 | 0 | 1 | | 0 | |  |
| Case 49 | 210 | 1 | 1 | | 1 | | 1 | | 1 | | 1 | | 1 | | 1 | | | 0 | 1 | 1 | | 1 | |  |
| Case 50 | 90 | 0 | 0 | | 0 | | 0 | | 0 | | 0 | | 1 | | 0 | | | 0 | 1 | 0 | | 0 | |  |
| Case 51 | 270 | 1 | 0 | | 1 | | 1 | | 1 | | 0 | | 0 | | 0 | | | 0 | 1 | 0 | | 0 | |  |
| Case 52 | 180 | 1 | 1 | | 1 | | 0 | | 1 | | 0 | | 0 | | 0 | | | 0 | 1 | 0 | | 0 | |  |
| Case 53 | 240 | 1 | 0 | | 1 | | 0 | | 1 | | 0 | | 1 | | 0 | | | 0 | 1 | 0 | | 0 | |  |
| Case 54 | 270 | 0 | 0 | | 0 | | 1 | | 0 | | 1 | | 1 | | 1 | | | 0 | 1 | 1 | | 0 | |  |
| Case 55 | 270 | 1 | 0 | | 1 | | 1 | | 1 | | 0 | | 1 | | 0 | | | 0 | 1 | 0 | | 0 | |  |
| Case 56 | 255 | 1 | 1 | | 1 | | 1 | | 1 | | 1 | | 1 | | 0 | | | 1 | 0 | 1 | | 1 | |  |
| Case 57 | 240 | 1 | 0 | | 1 | | 1 | | 0 | | 0 | | 1 | | 1 | | | 1 | 0 | 0 | | 0 | |  |
| Case 58 | 240 | 1 | 0 | | 1 | | 1 | | 1 | | 1 | | 1 | | 0 | | | 1 | 1 | 1 | | 1 | |  |
| Case 59 | 50 | 0 | 0 | | 1 | | 1 | | 0 | | 0 | | 1 | | 0 | | | 0 | 1 | 0 | | 0 | |  |
| Case 60 | 285 | 1 | 1 | | 1 | | 1 | | 1 | | 1 | | 0 | | 0 | | | 0 | 0 | 1 | | 0 | |  |
| Case 61 | 270 | 1 | 0 | | 1 | | 1 | | 1 | | 0 | | 1 | | 0 | | | 1 | 1 | 1 | | 0 | |  |
| Case 62 | 150 | 1 | 1 | | 1 | | 1 | | 1 | | 0 | | 1 | | 0 | | | 0 | 1 | 1 | | 1 | |  |
| Case 63 | 270 | 1 | 1 | | 1 | | 1 | | 1 | | 1 | | 1 | | 0 | | | 0 | 1 | 0 | | 1 | |  |
| Case 64 | 270 | 1 | 0 | | 1 | | 1 | | 1 | | 1 | | 1 | | 0 | | | 0 | 1 | 1 | | 0 | |  |
| Case 65 | 180 | 1 | 1 | | 0 | | 0 | | 1 | | 0 | | 1 | | 0 | | | 0 | 1 | 0 | | 1 | |  |
| Case 66 | 120 | 1 | 1 | | 1 | | 0 | | 1 | | 1 | | 1 | | 0 | | | 0 | 1 | 0 | | 0 | |  |
| Case 67 | 240 | 1 | 0 | | 0 | | 1 | | 1 | | 1 | | 1 | | 1 | | | 0 | 0 | 0 | | 0 | |  |
| Case 68 | 140 | 1 | 0 | | 1 | | 1 | | 1 | | 0 | | 0 | | 1 | | | 0 | 1 | 0 | | 0 | |  |
| Case 69 | 285 | 1 | 0 | | 1 | | 1 | | 1 | | 1 | | 1 | | 0 | | | 1 | 1 | 1 | | 0 | |  |
| Case 70 | 240 | 1 | 0 | | 1 | | 1 | | 1 | | 0 | | 1 | | 0 | | | 0 | 0 | 1 | | 1 | |  |
| Case 71 | 140 | 1 | 0 | | 1 | | 0 | | 0 | | 1 | | 1 | | 0 | | | 0 | 0 | 0 | | 0 | |  |
| Case 72 | 270 | 1 | 1 | | 1 | | 0 | | 1 | | 1 | | 1 | | 1 | | | 0 | 1 | 0 | | 0 | |  |
| Case 73 | 40 | 1 | 1 | | 1 | | 0 | | 1 | | 0 | | 1 | | 0 | | | 0 | 1 | 0 | | 0 | |  |
| Case 74 | 270 | 1 | 0 | | 0 | | 1 | | 0 | | 0 | | 1 | | 1 | | | 0 | 1 | 0 | | 0 | |  |
| Case 75 | 270 | 1 | 0 | | 1 | | 1 | | 1 | | 1 | | 1 | | 0 | | | 1 | 1 | 0 | | 0 | |  |
| Case 76 | 160 | 1 | 0 | | 0 | | 0 | | 1 | |  | | 1 | | 0 | | | 0 | 1 | 0 | | 0 | |  |
| Case 77 | 100 | 0 | 0 | | 1 | | 1 | | 1 | | 0 | | 1 | | 0 | | | 1 | 1 | 1 | | 0 | |  |
| Case 78 | 0 | 1 | 0 | | 1 | | 1 | | 1 | | 1 | | 1 | | 1 | | | 0 | 1 | 0 | | 0 | |  |
| Case 79 | 240 | 1 | 0 | | 1 | | 1 | | 1 | | 0 | | 1 | | 0 | | | 0 | 1 | 0 | | 0 | |  |
| Case 80 | 210 | 1 | 1 | | 1 | | 0 | | 1 | | 1 | | 1 | | 1 | | | 0 | 0 | 0 | | 0 | |  |
| Case 81 | 190 | 0 | 0 | | 1 | | 1 | | 1 | | 1 | | 1 | | 0 | | | 0 | 0 | 0 | | 1 | |  |
| Case 82 | 210 | 0 | 1 | | 1 | | 1 | | 1 | | 0 | | 1 | | 1 | | | 0 | 1 | 1 | | 0 | |  |
| Case 83 | 160 | 1 | 0 | | 1 | | 0 | | 1 | | 0 | | 1 | | 1 | | | 1 | 1 | 0 | | 0 | |  |
| Case 84 | 270 | 0 | 0 | | 1 | | 1 | | 0 | | 0 | | 1 | | 0 | | | 1 | 0 | 1 | | 0 | |  |
| Case 85 | 120 | 1 | 1 | | 1 | | 0 | | 1 | | 0 | | 0 | | 0 | | | 0 | 1 | 1 | | 0 | |  |
| Case 86 | 150 | 1 | 1 | | 1 | | 0 | | 1 | | 0 | | 0 | | 1 | | | 1 | 1 | 0 | | 0 | |  |
| Case 87 | 120 | 1 | 0 | | 1 | | 1 | | 0 | | 0 | | 0 | | 0 | | | 0 | 1 | 0 | | 0 | |  |
| Case 88 | 240 | 0 | 0 | | 1 | | 1 | | 1 | | 1 | | 1 | | 0 | | | 0 | 0 | 0 | | 0 | |  |
| Gender: male, 1; female, 2; | | | | | | |  | |  | |  | |  | |  | | |  |  |  | |  | |  |
| Age: ≥50, 1; <50, 0 | | |  | |  | |  | |  | |  | |  | |  | | |  |  |  | |  | |  |
| HBsAg: positive, 1; negative, 0 | | | | | | | | | | |  | |  | |  | | |  |  |  | |  | |  |
| AFP positive, 1; negative, 0 | | | | | | |  | |  | |  | |  | |  | | |  |  |  | |  | |  |
| Cirrhosis: yes, 1; no, 0 | | | | |  | |  | |  | |  | |  | |  | | |  |  |  | |  | |  |
| Tumor grade: I-II,0; III-IV,1 | | | | | | |  | |  | |  | |  | |  | | |  |  |  | |  | |  |
| Tumor size: ≥5, 1; <5, 0 | | | | |  | |  | |  | |  | |  | |  | | |  |  |  | |  | |  |
| Tumor #: single, 0; multiple, 1 | | | | | | |  | |  | |  | |  | |  | | |  |  |  | |  | |  |
| Embolus: yes, 1; no, 0 | | | | |  | |  | |  | |  | |  | |  | | |  |  |  | |  | |  |
| Capsule: yes, 1; no, 0 | | | | |  | |  | |  | |  | |  | |  | | |  |  |  | |  | |  |
| Recurrence: yes, 1; no, 0 | | | | |  | |  | |  | |  | |  | |  | | |  |  |  | |  | |  |
| Metastasis: yes, 1; no, 0 | | | | |  | |  | |  | |  | |  | |  | | |  |  |  | |  | |  |
